# Somatic BRCA1 mutations in clinically sporadic breast cancer with medullary histological features

**DOI:** 10.1007/s00432-018-2609-5

**Published:** 2018-02-17

**Authors:** Markus Rechsteiner, Konstantin Dedes, Daniel Fink, Bernhard Pestalozzi, Bettina Sobottka, Holger Moch, Peter Wild, Zsuzsanna Varga

**Affiliations:** 1 Department of Pathology and Molecular Pathology, University Hospital Zurich, 8091 Zurich, Switzerland; 2 Department of Gynecology, University Hospital Zurich, Zurich, Switzerland; 3 Clinic for Oncology, University Hospital Zurich, Zurich, Switzerland

**Keywords:** Sporadic breast cancer, Medullary features, BRCA1/2 mutation, NGS

## Abstract

**Background:**

The role of somatic *BRCA1*/*2* gene mutations in breast cancer is getting increasing attention in view of hereditary disease. The medullary phenotype and triple negative intrinsic subtypes are often, but not exclusively encountered in *BRCA1* germline mutated breast cancer, whilst for *BRCA2*, no association to specific histological features are known. In this study, we addressed the relationship between morphological medullary phenotype and *BRCA1*/*2* somatic mutations in breast cancer without known positive family anamnesis.

**Methods:**

32 clinically sporadic breast cancers with medullary features were analyzed for somatic *BRCA1*/*2* mutations (all coding exons) with next-generation sequencing technology. Paraffin-embedded formalin-fixed breast cancer samples from all patients were analyzed.

**Results:**

Three of 32 tumors (9%) had pathogenic (ARUP class-5) *BRCA1* gene alterations. Two of these pathogenic variants exhibited deletions leading to frameshift mutations (p.Glu23fs, p.Val1234fs), and the remaining single-nucleotid-variant resulted in premature STOP codon (p.Glu60Ter). In one patient, the same pathogenic *BRCA1* mutation was detected (p.Glu23fs) in normal breast tissue. Retrospective follow-up in two patients revealed a positive family history for breast cancer and consecutive germline mutation testing confirmed presence of *BRCA1* mutations. No somatic pathogenic *BRCA2* mutations were detected.

**Conclusions:**

*BRCA1* mutation testing may be useful in clinically sporadic breast cancer patients with medullary features to identify potential mutation carriers independently from intrinsic molecular subtype. Formalin-fixed paraffin-embedded cancer tissue can undergo testing within a routine molecular-diagnostic setting as a clinical *BRCA1*/*2* mutation screening strategy.

## Introduction

Assessment of BRCA1/2 gene mutation status from formalin-fixed paraffin-embedded (FFPE) tissue became a routine procedure for patients with high-grade serous ovarian cancer, as patients with evidence of such mutations are eligible for therapies including the PARP inhibitor olaparib (Hennessy et al. 2010; Mafficini et al. 2016; Moschetta et al. 2016; Muggia 2009; Oza et al. 2015). The role of BRCA1/2 gene mutations in patients with breast cancer is also getting more attention, with genetic counseling in view of a hereditary disease becoming a highly demanding field in patient care (Farrugia et al. 2008; Gonzalez-Angulo et al. 2011; Gross et al. 2016; Kwon et al. 2010a, b). The majority of *BRCA1* mutated breast cancers are so-called “triple negative” or of “basal-type”. In contrast, not all “triple negative” breast cancer patients have a germline *BRCA1*/*2* mutation. The indication for BRCA1/2 mutation testing is mainly based on clinical criteria, other than on histomorphological features (Dabbs 2012; Lakhani et al. 2012; Lips et al. 2017).

The medullary phenotype of breast cancer, which is often but not exclusively encountered in *BRCA1* germline mutation carriers, cannot reliably be used as an indication for genetic testing. (Dabbs 2012; Lakhani et al. 2012; Lips et al. 2017). The current WHO classification on breast cancer defines medullary differentiation as invasive high-grade carcinomas exhibiting various amounts of lymphocytic infiltration typically lacking in situ components and showing sharply demarcated edges towards the tumor periphery (Dabbs 2012; Lakhani et al. 2012; Lips et al. 2017). Overall survival of typical and atypical medullary breast carcinomas seem to be quite similar to each other, however, prognostic difference to ductal non-special type (NST) breast cancer is controversially reported in the literature (Dabbs 2012; Lakhani et al. 2012; Lips et al. 2017) (Mateo et al. 2016; Mavaddat et al. 2012). In case of *BRCA2* mutations in breast cancer, suggestive morphological and prognostic features are even more unspecific and thus less helpful. A wide range of histological subtypes, mainly a luminal hormone receptor positive phenotype, can be seen in breast cancer patients with *BRCA2* germline mutation (Dabbs 2012; Lakhani et al. 2012; Lips et al. 2017).

**Fig. 1:**
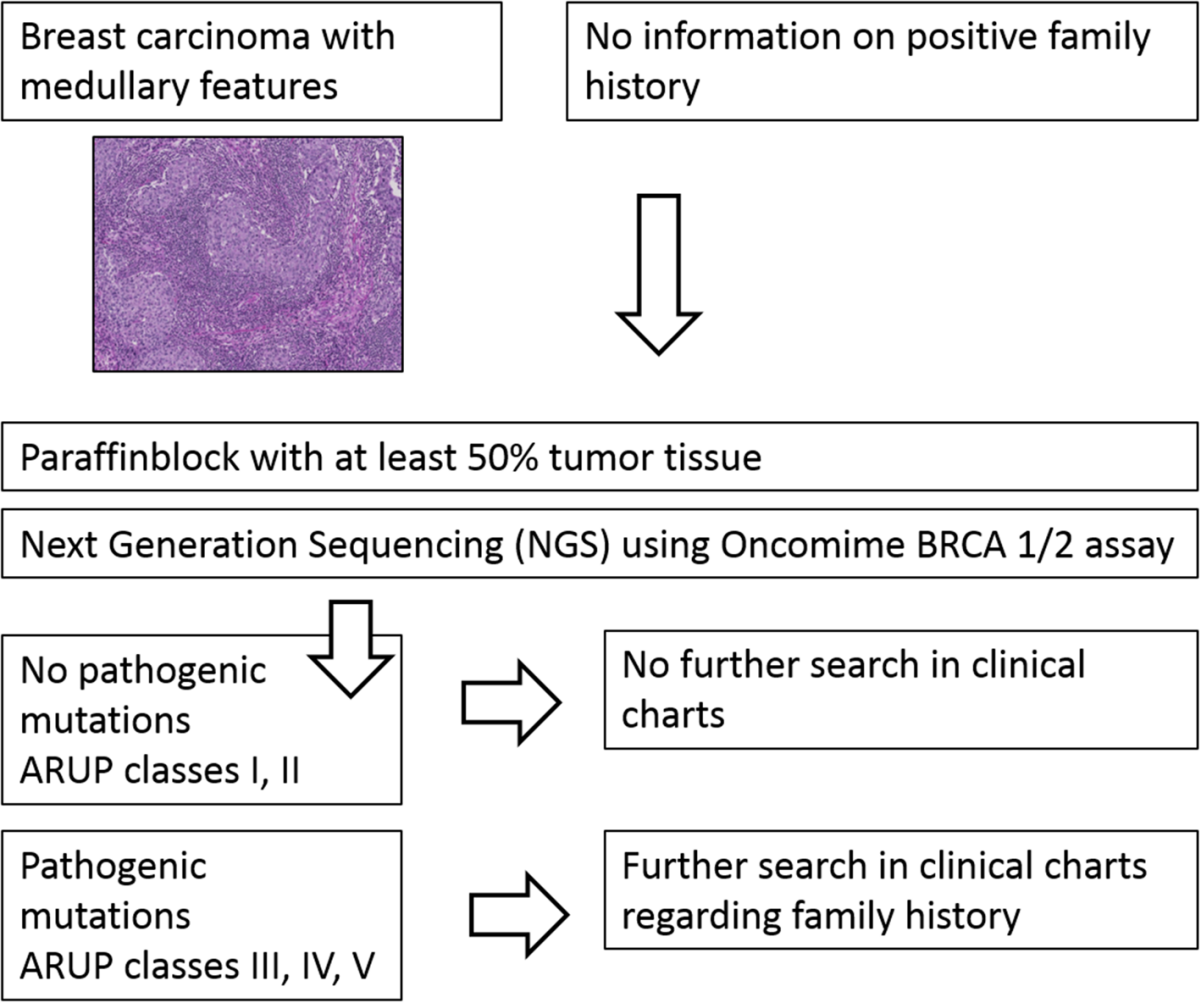
Flow chart of study design

In this study, we explored the relationship between a medullary phenotype in a series of breast cancer patients without known positive family history and *BRCA1*/*2* mutation status with next-generation sequencing (NGS) technology. We analyzed *BRCA1*/*2* mutation status in 32 breast cancer patients with medullary features (Fig. 1). The thus found pathogenic mutations were retrospectively compared with non-tumorous tissue and/or available long-term follow-up data.

## Materials and methods

### Breast cancer patients

**Fig. 2:**
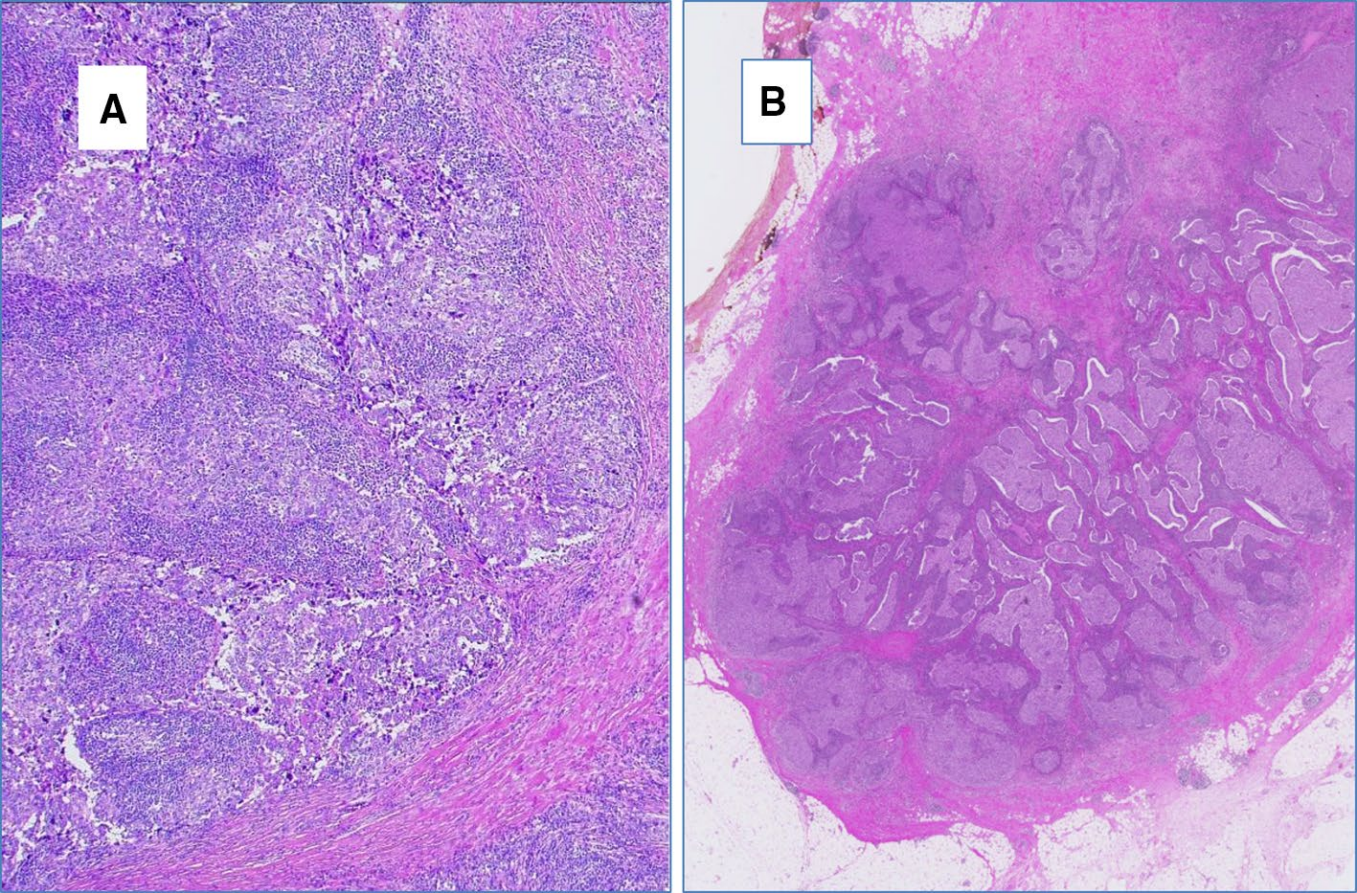
Histological appearance of breast carcinoma with medullary features. **a, b** Low-power view of an invasive breast cancer with medullary features showing abundant lymphocytic infiltration, serpentine-syntitial like tumor cell formation and sharp demarcation to tumor periphery, hematoxylin and eosin stain

**Fig. 3:**
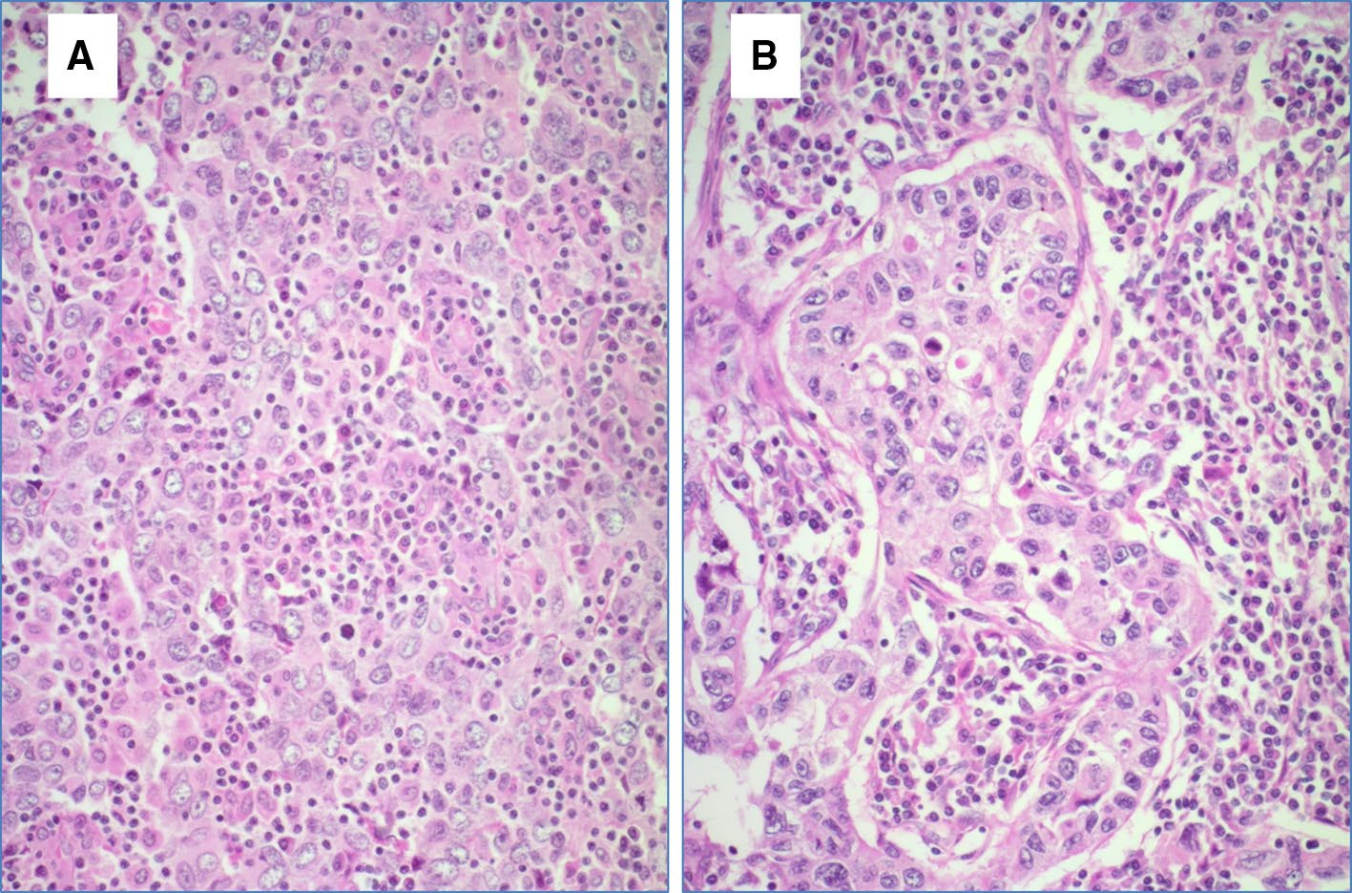
**a, b** High-power view of an invasive breast cancer with medullary features. Syntitial tumor cell formation exhibiting high nuclear polymorphism and mitotic figures. Hematoxylin and eosin stain

All tissue samples were retrospectively retrieved from the archives of the Department of Pathology and Molecular Pathology, University Hospital Zurich, Switzerland, encompassing a period of 1994–2015. We identified 32 breast cancer cases displaying medullary histological features as defined in the WHO 2012 as follows: all tumors had some or all of the following features as sharp circumscription or pushing peripheral areas, syncytial growth pattern, mainly high-grade nuclear morphology and at least focal prominent stromal and intratumoral lymphocytic infiltration (Figs. 2, 3). Patients’ age varied from 31 to 85 years (mean age 52.3 years). Tumor size varied from 1.2 to 6.5 cm (mean tumor size 2.44 cm). 16 of 32 cases (50%) were negative for estrogen and progesterone receptors and also for Her2 (triple negative intrinsic phenotype), 3 of 32 cases (9.3%) were Her2 positive and 13 of 32 cases (40.7%) were hormone receptor positive and Her2 negative. All patients underwent either mastectomy or local wide excision with axillary lymph node dissection (Table 1). There was no history of ovarian cancer in this cohort, three patients had benign ovarian cysts including also one mature teratoma.

**Table 1:** Clinico-pathological features of the cohort

Case number	Histological diagnosis	Age (years)	Grading	Tumor size (cm)	ER	PR	HER2 IHC	HER2 FISH
1	NST with medullary features	39	G3	2.4	100% positive	100% positive	Score 0	Not amplified
2	NST with medullary features	85	G3	1.7	5% positive	5% positive	NA	Not amplified
3	NST with medullary features	57	G3	1.9	Negative	Negative	Score 0	NA
4	NST with medullary features	50	G3	2.2	Negative	Negative	Score 0	NA
5	NST with medullary features	52	G3	1.2	Negative	Negative	NA	Not amplified
6	NST with medullary features	39	G3	4	Negative	Negative	NA	Not amplified
7	NST with medullary features	31	G3	2	Negative	Negative	IHC 0	NA
8	NST with medullary features	53	G3	1.2	Negative	Negative	Score 3+	Amplified
9	NST with medullary features	69	G3	2.6	Negative	Negative	NA	NA
10	NST with medullary features	59	G3	2.6	Negative	Negative	NA	NA
11	NST with medullary features	49	G3	1.2	Negative	Negative	NA	Not amplified
12	NST with medullary features	60	G3	1.2	1% positive	1% positive	NA	Not amplified
13	NST with medullary features	49	G3	2.6	5% positive	5% positive	NA	Not amplified
14	NST with medullary features	50	G3	1	Negative	Negative	NA	Not amplifed
15	NST with medullary features	48	G3	2,5	60% positive	2% positve	Score 1+	Not amplified
16	NST with medullary features	65	G3	2.2	80% positive	Negative	Score 1+	Not amplified
17	NST with medullary features	49	G3	0.9	Negative	Negative	Score 0	Not amplified
18	NST with medullary features	36	G3	4.5	Negative	Negative	Score 0	Not ampified
19	NST with medullary features	56	G3	2.3	100% positive	10% positive	Score 1+	Not amplified
20	NST with medullary features	43	G3	2.2	100% positive	100% positive	Score 2+	Not amplified
21	NST with medullary features	45	G3	3.5	100% positive	50% positive	Score 2+	Not amplified
22	NST with medullary features	44	G3	6.5	Negative	Negative	Score 1+	Not amplified
23	NST with medullary features	65	G3	6	100% positive	90% positive	Score 1+	Not amplified
24	NST with medullary features	73	G3	5	100% positive	80% positive	Score 2+	Not amplified
25	NST with medullary features	55	G3	1.2	Negative	Negative	Score 0	NA
26	NST with medullary features	36	G3	3	20% positive	10% positive	Score 0	Not amplified
27	NST with medullary features	56	G3	2.2	100% positive	20% positive	Score 1+	Not amplified
28	NST with medullary features	45	G3	2.6	100% positive	80% positive	Score 3+	Amplified
29	NST with medullary features	54	G3	1.2	Negative	negative	Score 3+	Amplified
30	NST with medullary features	76	G3	0.6	Negative	Negative	Score 2+	Not amplified
31	NST with medullary features	47	G3	2.2	100% positive	Negative	Score 0	Not amplified
32	NST with medullary features	39	G3	1.8	Negative	Negative	Score 0	Not amplified

The study is a part of a retrospective larger breast cancer study previously approved by the Ethical Committee of the Canton Zurich (KEK-ZH-2012-553). For selected cases, required by the ethical approval, informed consents were obtained. All cases enrolled into the study cohort were anonymized for the study.

### Determination of hormone receptors and Her2 status

The hormone receptors (estrogen, ER and progesterone, PR) were determined in all cases using routine antibodies and pretreatment conditions. HER2 status was determined using the DAKO Herceptest, the Ventana CB11 and Ventana 45A antibodies. Manual pretreatment protocols or semi-automatic and automatic benchmark systems have been used. Detailed technologies for ER/PR and HER2 status evaluation have been published, previously (Varga et al. 2013, 2014).

### Next-generation sequencing (NGS)

For all NGS assays, representative cancer areas for the microdissection and DNA isolation were selected and marked by Z.V. on a freshly cut hematoxylin-eosin (HE) section. The marked tumor area was punched (length 2–4 mm, diameter 0.6 mm) from the paraffin block and DNA was isolated using a Promega DNA purification kit (Promega, Wisconsin, USA). Isolated genomic DNA was quantified using a fluorometric assay (Qubit, Thermo Fisher Scientific, Massachusetts, USA) and NGS libraries were amplified with the AmpliSeq Library Kit 2.0 and the Oncomine BRCA Assay (Thermo Fisher Scientific). Clonal amplification und sequencing was performed with Hi-Q chemistry on the Ion PGM platform according to manufacturer’s requirements (Thermo Fisher Scientific). NGS data was analyzed with the Torrent Suite v5.0.3 and the Ion Reporter v5.0 including the Oncomine BRCA workflow. NGS run metrics (on target reads, mean depth, uniformity) are summarized in Table 2. NGS reads were aligned to the reference genome hg19/GRCh37 and the transcripts for BRCA1 (NM_007300.3) and BRCA2 (NM_000059.3). The detected variants were filtered to the coding exons and excluded if they were listed in the commonSNP database (minor allele frequency > 1%). Additionally, variants were excluded if they had variant allele frequencies < 4% and variant allele coverage < 50× (internal validation of sensitivity of AmpliSeq assays). To further validate the performance of the Oncomine BRCA panel, a mixing dilution experiment was performed confirming the sensitivity of the assay for SNVs and INDELs. After filtering, the variants were annotated to the COSMIC, dbSNP, and ARUP BRCA databases (http://arup.utah.edu/database/BRCA/). ARUP classification was done according to Plon et al. (2008): (1) (not pathogenic or of no clinical significance); (2) (likely not pathogenic or of little clinical significance); (3) (uncertain); (4) (likely pathogenic); and (5) (definitely pathogenic). To further classify the impact of unknown mutations on protein level, SIFT (http://sift.jcvi.org/) and PolyPhen (http://genetics.bwh.harvard.edu/pph/) were used.

**Table 2:** Next-generation sequencing (NGS) run metrics (on target reads, mean depth, uniformity)

RV case	Gene	Transkript	Nucleotid change	Aminoacid change	Exon	Mutation ratio	ClinVar ID	ClinVar class	ARUP class	dbSNP	On target	Coverage	Uniformity
1	–	–	–	–	–	–	–	–	–	–	97.96	7175	94.11
2	–	–	–	–	–	–	–	–	–	–	97.93	3246	99.21
3	–	–	–	–	–	–	–	–	–	–	97.05	2611	98.03
4	–	–	–	–	–	–	–	–	–	–	97.64	2940	99.21
5	–	–	–	–	–	–	–	–	–	–	98.1	3226	99.21
6	BRCA1	NM_007300.3	c.3700_3704del GTAAA	p.Val1234fs	11	0.83	37542	pathogenic	5	rs80357609	97.42	1637	99.19
7	BRCA1	NM_007300.3	c.178C>T	p.Gln60Ter	5	0.54	54349	pathogenic	5	rs80357471	97.74	2892	99.21
8	–	–	–	–	–	–	–	–	–	–	94.49	1139	79.75
9	–	–	–	–	–	–	–	–	–	–	98.49	2347	98.7
10	–	–	–	–	–	–	–	–	–	–	97.5	1629	98.66
11	–	–	–	–	–	–	–	–	–	–	98.74	4121	98.28
12	–	–	–	–	–	–	–	–	–	–	98.51	1837	99.21
13	–	–	–	–	–	–	–	–	–	–	98.56	1962	99.48
14	BRCA2	NM_000059.3	c.7469T>C, c.7960C>G	p.Ile2490Thr, p.Leu2654Val	15, 17	0.98, 0.04	96852,–	1 likely benign; 1 uncertain significance,–	–	rs11571707,–	96.71	1680	98.47
15	–	–	–	–	–	–	–	–	–	–	97.68	1991	99.18
16	–	–	–	–	–	–	–	–	–	–	98.42	2323	99.21
17	BRCA2	NM_000059.3	c.9976A>T	p.Lys3326Ter	27	0.54	38266	benign	1	rs11571833	98.03	1840	98.69
18	BRCA1	NM_007300.3	c.68_69delAG	p.Glu23fs	2	0.56	–	pathogenic	5	–	98.14	1521	99.2
19	–	–	–	–	–	–	–	–	–	–	98.22	1764	99.09
20	–	–	–	–	–	–	–	–	–	–	98.17	1521	99.21
21	BRCA1	NM_007300.3	c.2584A>G	p.Lys862Glu	11	0.77	37476	benign	1	rs80356927	98.39	1633	99.48
22	BRCA2	NM_000059.3	c.4258G>T	p.Asp1420Tyr	11	0.37	41549	benign	1	rs28897727	98.48	2193	94.88
23	–	–	–	–	–	–	–	–	–	–	98.4	2214	90.58
24	–	–	–	–	–	–	–	–	–	–	98.75	1936	95.47
25	BRCA2	NM_000059.3	c.4258G>T	p.Asp1420Tyr	11	0.45	41,549	benign	1	rs28897727	97.4	2348	99.14
26	–	–	–	–	–	–	–	–	–	–	97.85	2177	99.35
27	BRCA1	NM_007300.3	c.2521C>T	p.Arg841Trp	11	0.53	17,681	benign	1	rs1800709	92.79	743	91.72
28	–	–	–	–	–	–	–	–	–	–	97.69	1741	99.07
29	–	–	–	–	–	–	–	–	–	–	98.36	2400	99.2
30	–	–	–	–	–	–	–	–	–	–	96.94	1937	99.47
31	–	–	–	–	–	–	–	–	–	–	97.92	1673	99.41
32	–	–	–	–	–	–	–	–	–	–	98.15	1296	99.16

## Results

Three of 32 cases (9%) had definitely pathogenic germline *BRCA1* mutations (ARUP class 5) which lead either to a frameshift or a STOP codon in the protein (Table 3). No pathogenic somatic BRCA2 mutations were observed.

**Table 3:** Pathogenicitiy classification of detected mutation in the analyzed 32 cases

RV case	I	II	III	IV	V
1	–	–	–	–	–
2	–	–	–	–	–
3	–	–	–	–	–
4	–	–	–	–	–
5	–	–	–	–	–
6	–	–	–	–	BRCA1: p.Val1234fs
7	–	–	–	–	BRCA1: p.Gln60Ter
8	–	–	–	–	–
9	–	–	–	–	–
10	–	–	–	–	–
11	–	–	–	–	–
12	–	–	–	–	–
13	–	–	–	–	–
14	–	BRCA2: p.Ile2490Thr	–	BRCA2: p.Leu2654Val	–
15	–	–	–	–	–
16	–	–	–	–	–
17	BRCA2: p.Lys3326Ter	–	–	–	–
18	–	–	–	–	BRCA1: p.Glu23fs
19	–	–	–	–	–
20	–	–	–	–	–
21	BRCA1: p.Lys862Glu	–	–	–	–
22	BRCA2: p.Asp1420Tyr	–	–	–	–
23	–	–	–	–	–
24	–	–	–	–	–
25	BRCA2: p.Asp1420Tyr	–	–	–	–
26	–	–	–	–	–
27	BRCA1: p.Arg841Trp	–	–	–	–
28	–	–	–	–	–
29	–	–	–	–	–
30	–	–	–	–	–
31	–	–	–	–	–
32	–	–	–	–	–

The first patient (no. 6) was diagnosed with breast cancer at an age of 40 years. NGS-based *BRCA1*/*2* testing showed a 5-basepair deletion (c.3700_3704delGTAAA) in exon 11 in the *BRCA1* gene which lead to a frameshift at aminoacid position 1234 (p.Val1234fs). The mutation is registered in the databases ARUP, ClinVar (ID 37542) and dbSNP (rs80357609) to be pathogenic. A second independent NGS library was prepared and sequenced which successfully validated the *BRCA1* mutation p.Val1234fs. Retrospective follow-up search revealed a positive family history in this patient. Due to a contralateral breast cancer 20 years after the initial diagnosis, the patient underwent germline *BRCA1* mutation testing, revealing the same pathogenic mutation in *BRCA1*.

The second patient (no. 7) with a pathogenic *BRCA1* alteration had a nonsense mutation (c.178C>T) in exon 5 in the *BRCA1* gene leading to a STOP codon in the protein (p.Gln60Ter). This mutation is as listed as well in the databases ARUP, ClinVar (ID 54349) and dbSNP (rs80357471) to be pathogenic. An independent NGS library was prepared and sequenced which successfully validated the mutation. Subsequent germline *BRCA1* mutation testing showed the same pathogenic mutation in *BRCA1*.

The third patient (no. 18) had as a deletion (c.68_69delAG) in the *BRCA1* gene, leading to a frameshift in the protein (p.Glu23fs). Subsequent NGS analysis of corresponding normal breast tissue revealed the same mutation, suggesting a hereditary disease. In this patient, there was no positive family history of breast cancer. Due to the young age (37 years at initial diagnosis) and the triple negative phenotype with medullary features, genetic counseling and testing was recommended to the patient at the weekly interdisciplinary tumor board. Four years after initial diagnosis, however, no records about germline *BRCA1*/*2* testing could be found.

**Fig. 4:**
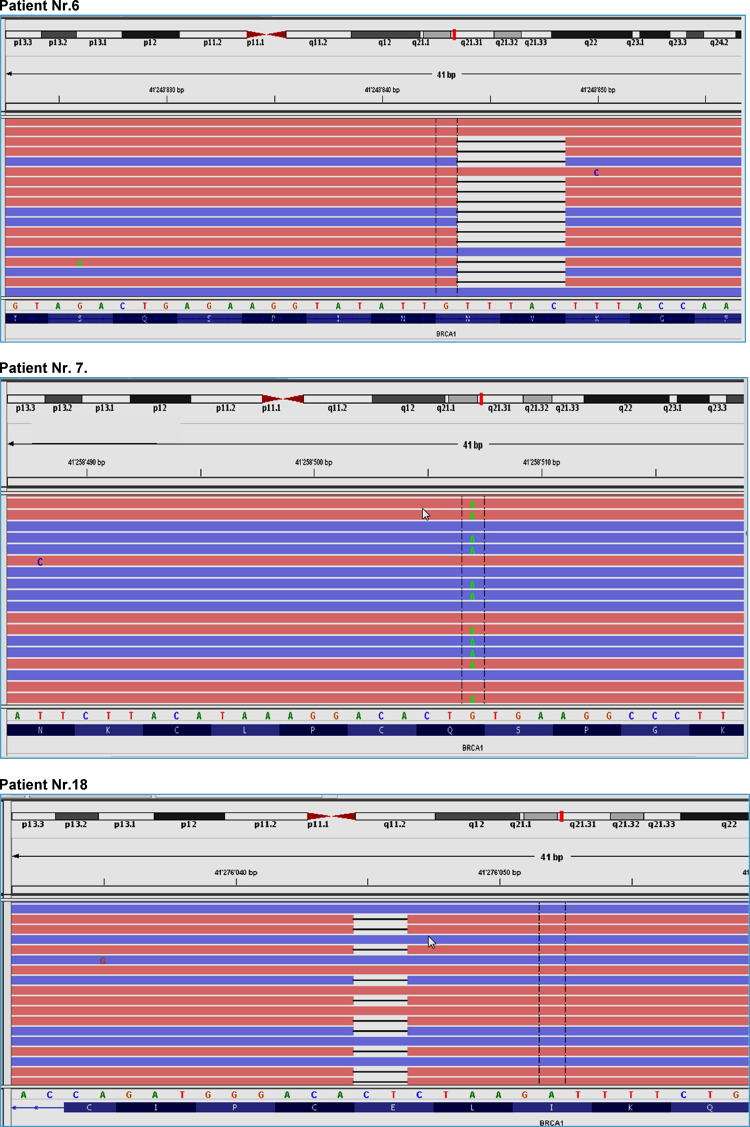
Pathogenic mutations of the BRCA1 gene in patients 6, 7 and 18

Pathogenic mutations of the BRCA1 gene are illustrated in details in Fig. 4.

In six patients, benign, likely benign or mutations of unknown significance (VUS) were detected (ARUP class 1–3).

In one patient (no. 14), two SNVs were detected in exon 15 (p.Ile2490Thr) and 17 (p.Leu2654Val) of the *BRCA2* gene. The mutation in exon 15 showed an allele frequency of almost 100%, representing a homozygous SNP, which was further underlined by a dbSNP entry (rs11571707) with a minor allele frequency (MAF) of 1.9% in the human population. The same mutation is registered in ClinVar (ID 96852) with conflicting interpretation of clinical significance. However, these entries are benign (11 entries), likely benign (1 entry), and of uncertain significance (1 entry), suggesting a non-pathogenic impact. The second mutation in patient no. 14 was detected at low allele frequency of 4%. Since this is at the limit of detection of our NGS system, a second independent NGS library was prepared and sequenced. The mutation p.Leu2654Val was successfully verified with an allele frequency of 5%. Interestingly, this mutation is registered neither in COSMIC, dbSNP nor in the ARUP. The protein alteration prediction tools SIFT and PolyPhen revealed highly destabilizing (damaging) values of 0 and 0.87, respectively. Therefore, the exon 17 mutation in *BRCA2* (p.Leu2654Val) was classified as VUS.

One nonsense mutation (patient no. 17) was detected at the 3-prime end in exon 27 of the *BRCA2* gene, leading to a STOP codon (p.Lys3326Ter). This mutation is registered in the ARUP and the ClinVar database and was suggested to be clinically not significant (ARUP class 1) (Farrugia et al. 2008; Tavtigian et al. 2008), and benign (ClinVar ID 38266), respectively. Additionally, the mutation is listed in the dbSNP database (rs11571833) with a minor allele frequency (MAF) of 0.4% in the human population. The mutation was not validated in an independent NGS library run, since it was assumed to be non-pathogenic.

Additional mutations which were assumed to be non-pathogenic according to ClinVar and ARUP entries were detected in patient no. 21 (*BRCA1*, p.Lys862Glu), no. 22 (*BRCA2*, p.Asp1420Tyr), no. 25 (*BRCA2*, p.Asp1420Tyr), and no. 27 (*BRCA1*, p.Arg841Trp). These mutations were not validated with an independent NGS run due to non-pathogenicity either.

## Discussion

We performed BRCA1/2 testing by next-generation sequencing in clinically sporadic breast cancer patients with medullary like breast cancer and without known *BRCA1* and *BRCA2* mutations at presentation. Our study demonstrates that about nine percent of medullary like breast cancer patients without any known positive family history were *BRCA1* gene mutation carriers, whereas no pathogenic somatic *BRCA1*/*2* mutations could be found.

*BRCA1*/*2* mutations regained clinical attention, as patients with high-grade serous ovarian carcinomas with pathogenic *BRCA1*/*2* mutations displayed improved overall and recurrence-free survival if treated with platinum-based therapy in combination with PARP inhibitors such as olaparib (Hennessy et al. 2010; Mafficini et al. 2016; Moschetta et al. 2016; Muggia 2009; Oza et al. 2015; Kwon et al. 2010; Muggia et al. 2011). Patients with *BRCA1*/*2* germline mutated serous ovarian cancers responded better to first-line chemotherapy in the metastatic setting in comparison with sporadic serous high-grade carcinomas (Hennessy et al. 2010; Mafficini et al. 2016; Moschetta et al. 2016; Muggia 2009; Oza et al. 2015; Kwon et al. 2010; Muggia et al. 2011). Furthermore, resistance to taxane containing regimens have been documented in serous high-grade ovarian carcinomas displaying *BRCA1*/*2* germline mutations (Hennessy et al. 2010; Mafficini et al. 2016; Moschetta et al. 2016; Muggia 2009; Oza et al. 2015; Kwon et al. 2010; Muggia et al. 2011).

The role of pathogenic BRCA1/2 mutations in breast cancer is currently not linked to specific therapies and is rather restricted to the choice for genetic counseling (Chalasani and Livingston 2013). The need for genetic counseling including germ line BRCA1/2 testing in breast cancer patients is increasing, however, selection criteria remain mostly a positive family history additionally to breast cancers harboring a triple negative intrinsic phenotype (Kwon et al. 2010a, b; Chalasani and Livingston 2013). Specific histological signs only exist for germline mutations in *BRCA1*. Breast cancers arising in *BRCA1* germ line mutation carriers are mostly triple negative, of younger patients (< 50 years), high-grade and display so-called medullary features. Medullary-type breast cancers are characterized by dense lymphocytic infiltrate and pushing peripheral borders (Gonzalez-Angulo et al. 2011; Kwon et al. 2010a, b; Dabbs 2012; Lakhani et al. 2012; Chalasani and Livingston 2013). The frequency of BRCA1 gene allelic loss were reported to be more frequent in ER negative than in ER positive sporadic breast cancer cases (39 vs 12%) (Rhiem et al. 2010). In unselected triple negative breast cancers, around 19% mutations were reported in the BRCA1/2 genes including also scattered somatic mutations (15% in BRCA and 3.9% in BRCA2 genes) (Gonzalez-Angulo et al. 2011). Interestingly, there is lack of data on sporadic medullary carcinomas and its association with BRCA1 germline or somatic mutations. The 6.25% frequency of BRCA1 frame shift mutations in our study is lower than reported frequencies in unselected triple negative breast cancers or in sporadic cases or in serous high-grade ovarian carcinomas without family history varying from 19 to 28%) (Gonzalez-Angulo et al. 2011; Rhiem et al. 2010) (Mafficini et al. 2016; Moschetta et al. 2016). Whether this lower frequency is due to the relatively small number of cases in our cohort or to the fact that medullary phenotype is alone not pathognomic enough to predict BRCA1 mutation status, needs to be validated in further studies. On the other hand, 40.7% of the cases were hormone receptor positive in our study, which is unusually high in comparison to classical triple negative phenotype of classical medullary breast carcinoma. The high proportion of hormone receptor positive cases might possibly reflect the histological variability of medullary differentiation in breast cancer and might also contribute to the low frequency of somatic BRCA1 mutations.

In selected triple negative breast cancer cases, 57% were found to have BRCA1 and 23% BRCA2 germ line mutations (Gonzalez-Angulo et al. 2011).

The term BRCAness, defined as DNA repair loss in the BRCA1/2 genes without germ line mutations, resulting in the same function loss and inactivation the BRCA1/2 genes, has been conflictingly discussed and addressed in the literature (Muggia 2009; Lips et al. 2017; Muggia et al. 2011; Chalasani and Livingston 2013; Vollebergh et al. 2014). Probably, the choice of technology for assessing the DNA damage as MPLA, qPCR, IHC or aCGH methodologies resulted in non-standardized definitions of what consists of BRCAness damage (Chalasani and Livingston 2013; Lips et al. 2017). In two studies, breast cancers with BRCA1-like signature, as defined as BRCAness of the BRCA1 gene were found in 18% of breast cancers and showing a better response to anthacyline-based or platin containing high-dose chemotherapies (Lips et al. 2017; Vollebergh et al. 2014). One further study reported a higher frequency of BRCA2-like signatures in hormone receptor positive breast cancer cases and also a better response to neoadjuvant chemotherapy with anthracycline-based regiments (Lips et al. 2017). Which technology is the most reliable to predict clinical outcome or the indication to genetic counseling in cases with BRCAness evidence is not solved at the current time and needs clinical validation in further studies (Muggia 2009; Muggia et al. 2011; Chalasani and Livingston 2013).

The current technology of next-generation sequencing in paraffin-embedded, formalin-fixed material was first described in ovarian high-grade serious cancer both for somatic and germ line mutations, providing a sensitivity of > 90% after verifying the data with Sanger sequencing (Mafficini et al. 2016). This technology became meanwhile standard in somatic BRCA1/2 testing in ovarian cancer and was also the choice of methodology in our study. The classification system for detected mutations using NGS and Sanger sequencing was recently defined by Eccles (Eccles et al. 2015). A five-tier score system, ranging from non- or likely non-pathogenic mutations (classes 1/2) through uncertain significance as class 3 to likely or definitely pathogenic mutations (classes 4/5) is linked to recommendations in terms of clinical management and to assessing risk situation of the given patient (Eccles et al. 2015). Scores 4/5 are recommended as high-risk patient with appropriate genetic counseling, whilst classes 1 and 2 should follow management based on family history alone. At the current time, no clear guidelines exist for the clinical management of genes of uncertain significance (class 3). (Farrugia et al. 2008; Plon et al. 2008; Tavtigian et al. 2008) (Eccles et al. 2015). In or study, we found three definitely pathogenic somatic mutations of the BRCA1 genes (class 5).

Based on the data in our cohort, medullary breast cancer phenotype without known family history in breast cancer, has a low frequency of somatic BRCA1 mutations, even though these mutations turned out to correlate with germ line mutations of the BRCA1 gene independently from other factors as younger age and triple negative intrinsic phenotype. These data might be of help in case genetic counseling in sporadic breast cancers with medullary features. On the other hand, medullary phenotype without family anamnesis did not have any class 4 or 5 somatic mutations in the BRCA2 gene, pointing out to the role of the BRCA1 gene only in breast cancer with medullary features. Our data need to be validated in further lager studies.
